# *Sirt6 mRNA*-incorporated endothelial microparticles (EMPs) attenuates DM patient-derived EMP-induced endothelial dysfunction

**DOI:** 10.18632/oncotarget.23259

**Published:** 2017-12-15

**Authors:** Tong Jing, Kuang Ya-Shu, Wang Xue-Jun, Hou Han-Jing, Lai Yan, Yao Yi-An, Chen Fei, Liu Xue-Bo

**Affiliations:** ^1^ Department of Cardiology, Shanghai Tongji Hospital, Tongji University, Shanghai, China

**Keywords:** EMPs, *sirtuin 6*, endothelial dysfunction, diabetes, vascular complication

## Abstract

**Background:**

Endothelial microparticles (EMPs) are small vesicles released by endothelial cells (ECs); they are considered biomarkers for endothelial dysfunction and therapeutic targets in diabetes-related vascular disease. Sirtuins have also been shown to play important roles in diabetes by regulating endothelial dysfunction. However, the effect of sirtuin-incorporated EMPs on their parental ECs remains unknown.

**Aim:**

The present study aims to investigate the diagnostic value of EMPs in diabetes and detect the protective effects of *sirtuin 6* (*Sirt6*) *mRNA* -incorporated EMPs on endothelial dysfunction.

**Methods:**

EMPs were prepared from cultured HUVECs and venous blood from patients with diabetes (n=10) and from healthy volunteers (n=6) after sequential centrifugation. Adv-*Sirt6* or *Sirt6* siRNA was used to alter Sirt6 expression. EC angiogenesis, inflammatory phenotypes, nitric oxide (NO) formation and eNOS phosphorylation were used to evaluate endothelial dysfunction.

**Results:**

The levels of EMPs in diabetic patients and high glucose-cultured HUVECs are high, whereas Sirt6 expression in plasma and EMPs is low. EMPs generated from diabetic patients or high glucose-cultured HUVECs increase inflammatory chemokine release and blunt EC angiogenesis. Furthermore, EMPs enriched with *Sirt6* mRNA induces EC angiogenesis, increases eNOS phosphorylation and impedes inflammatory chemokine release. Inhibition of *Sirt6* mRNA expression in EMPs by siRNA hinders angiogenesis and eNOS phosphorylation but increases cellular inflammation.

**Conclusion:**

The *Sirt6 mRNA*-carrying EMPs may ameliorate endothelial dysfunction in diabetic patients.

## INTRODUCTION

The incidence of diabetes mellitus (DM) is prevalent in both developed and developing countries. As of 2035, an estimated 592 million individuals worldwide will be diagnosed with DM, which is increased from 387 million in 2014 [1]. DM-induced macrovascular and microvascular complications lead to reduced quality of life, added health care costs and even early death [2]. It is noteworthy that vascular endothelial dysfunction is the main factor in the pathogenesis of diabetic vascular complications via increased inflammation, dysregulated blood flow, cellular trafficking, decreased nitric oxide (NO) production and the induction of endothelial cell death, thus contributing to increased mortality rates [3, 4]. Consequently, the comprehensive analysis and amelioration of endothelial impairment may inhibit the progression of DM-related vascular complications.

Endothelial dysfunction is associated with inordinate intercellular information exchange between endothelial cells (ECs) and immune cells, smooth muscle cells, fibroblasts or even parent ECs [5]. In addition, intercellular information exchange may involve the release of extracellular vesicles, which can function as intercellular carriers of ligands, enzymes, RNA and miRNA [6]. However, the precise mechanism of intercellular information exchange between ECs in DM remains largely unknown.

Recently, documents have verified that endothelial microparticles (EMPs) are novel, complex membrane-shed vesicles that facilitate endothelial dysfunction in DM-related vascular complications [2]. EMPs range from 0.1-1 μm in size and are generated from ECs during apoptosis, activation or injury [7]. Importantly, previous data reveal that the biological content (protein, lipid, miRNA and mRNA) and functional effect of EMPs depend on the condition of the ECs from which they are released [8]. Hyperglycemic conditions significantly increase EMP expression, which is low under normal conditions, and subsequently change their biological effect on target cells [9]. Under DM conditions, microparticles (MPs) have shown noxious effects on ECs in a previous study [6, 7, 10]. *in vivo* studies have shown that endothelial cells or platelet derived-MPs impair angiogenesis, reduce cerebral microvascular density and accelerate the progression and severity of retinopathy in DM; meanwhile, MPs, especially CD31+ MPs, mediated EC dysfunction in *ex vivo* studies [10-12]. In addition, EMPs also act as vectors during intercellular information exchange though coupling with membrane-associated receptors, releasing signaling molecules, such as active proteins and miRNA, and finally, mediating adaptive responses [13], but the effects of EMPs on their parent ECs are still unclear. Therefore, the present study aims to understand the release levels of EMPs and they cause endothelial dysfunction in DM and high glucose mediated-HUVECs.

Sirtuins (Sirts), a family of evolutionarily conserved nicotinamide adenine dinucleotide-dependent histone deacetylases, contains 7 Sirt genes, which have been tested to participate in multiple pathophysiological processes, such as DNA damage repair, apoptosis, senescence and neurodegeneration [14]. Recently, mounting evidence suggests that Sirts exerts effects in multiple cardiovascular disease models, including atherosclerosis, cardiac hypertrophy, chronic inflammation and vascular aging [15]. Additionally, previous studies have revealed that Sirt6 expression is downregulated in response to DM. Moreover, Sirt6 deficiency exacerbates the DM-induced impairment of wound healing and endothelial senescence [16, 17]. However, Sirt6 expression in EMPs remains unknown. This study aims to investigate the possible roles of Sirt6-containing EMPs in endothelial dysfunction in DM.

## RESULTS

### Increased plasma EMPs levels and reduced Sirt6 expression in DM patients

**Figure 1:**
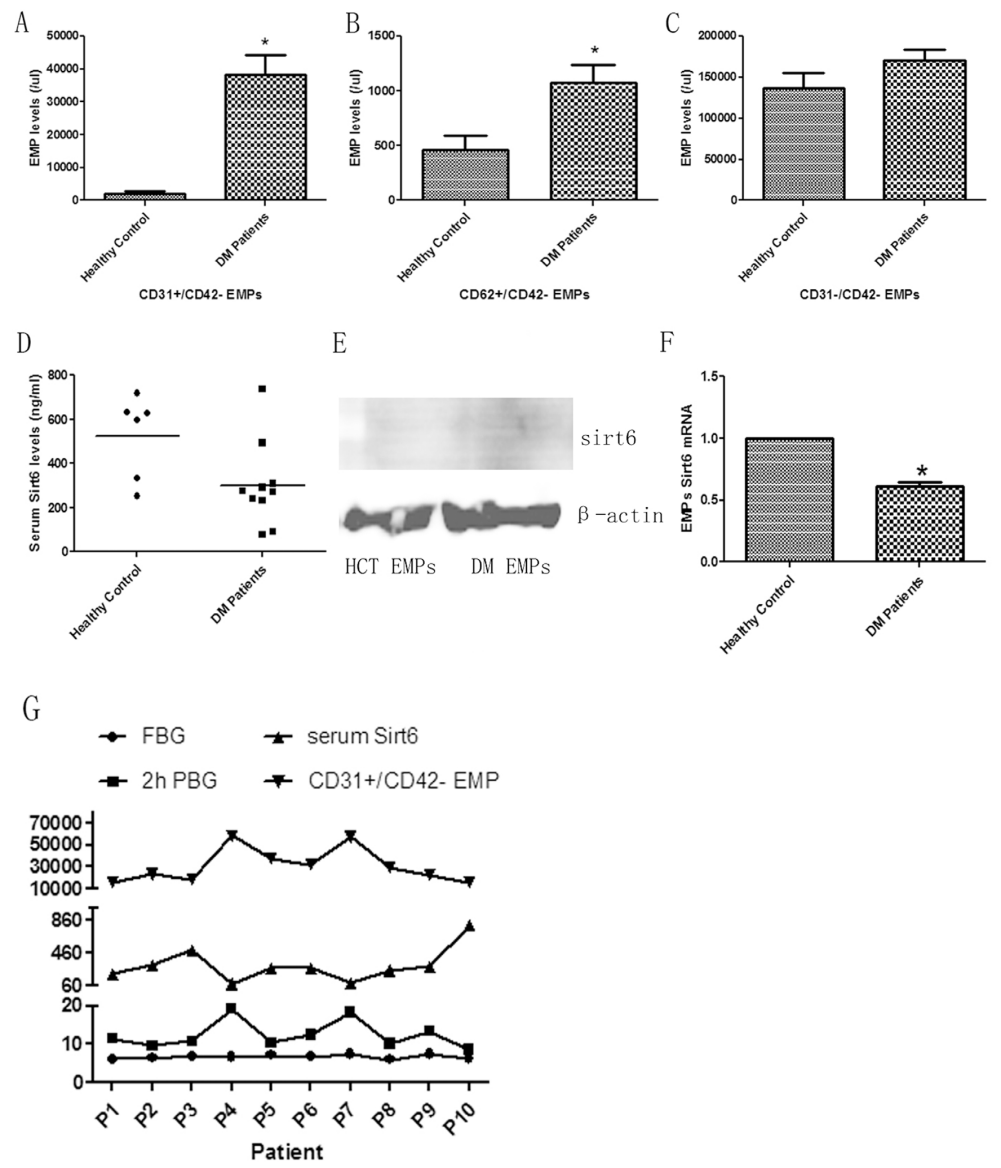
EMP levels and Sirt6 expression in DM patients **(A)** Increased apoptosis-derived EMP (CD31+) levels in DM patients compared to healthy controls. **(B)** Increased activated EMP (CD62+) levels in DM patients compared to healthy controls. **(C)** There is no difference in platelet-derived (CD42+) MPs between healthy controls and DM patients. **(D)** Decreased serum Sirt6 levels in DM patients. **(E)** There is no Sirt6 protein in EMPs. **(F)** Decreased incorporation of *Sirt6 mRNA* in DM patient-derived EMPs compared to EMPs from healthy controls. **(G)** Boosted CD31+ EMP levels related with decreased serum Sirt6 levels and increased 2-hour post-meal blood glucose levels. Healthy control, n=6; and DM, n=10. EMPs, endothelial microparticles; MPs, microparticles; HCT, healthy control; DM, diabetes mellitus; FBG, fast blood glucose; 2h PBG, 2-hour post-meal blood glucose; p(n), patient numble. ^*^ p<0.05. *GAPDH* mRNA serves as an internal control.

When compared with the healthy group, plasma CD31+/CD42- EMPs levels were significantly increased in the DM group (Figure 1A). Meanwhile, CD62+/CD42- EMP levels were augmented in DM patients (Figure 1B). However, the levels of CD42+ MPs were not significantly different between the two groups (Figure 1C). This indicated the increased release of EMPs into plasma in DM patients.

Decreased expression of Sirt6 is a widely accepted risk factor of DM and atherosclerosis. Therefore, we speculated that EMP-mediated injury may be caused by Sirt6 deficiency. In this study, we first detected the expression of plasma Sirt6 using ELISA. The results revealed that Sirt6 expression in plasma from DM patients was significantly reduced compared with plasma from healthy volunteers (Figure 1D and Supplementary Figure 1A). Then, we measured Sirt6 mRNA or protein expression in EMPs. In contrast to *Sirt6 mRNA*, immunoblot experiments revealed nearly no expression of Sirt6 protein in different EMPs (Figure 1E), suggesting that *Sirt6 mRNA* is selectively packaged in EMPs. Additionally, we noted that *Sirt6 mRNA*-containing EMPs were obviously decreased in DM patients (Figure 1F). These data indicated that Sirt6-containing EMPs may play important roles in DM patients.

To estimate the EMPs and plasma Sirt6 levels whether related with blood glucose levels, we examined the blood glucose levels in DM patients and found that there was significant difference in 2-hour post-meal blood glucose but not in fasting blood-glucose. Additionally, the increased EMP levels and decreased Sirt6 levels were observed in patients with high blood-glucose levels accordingly (Figure 1G).

### EMPs from DM patients induced EC dysfunction *ex vivo*

**Figure 2:**
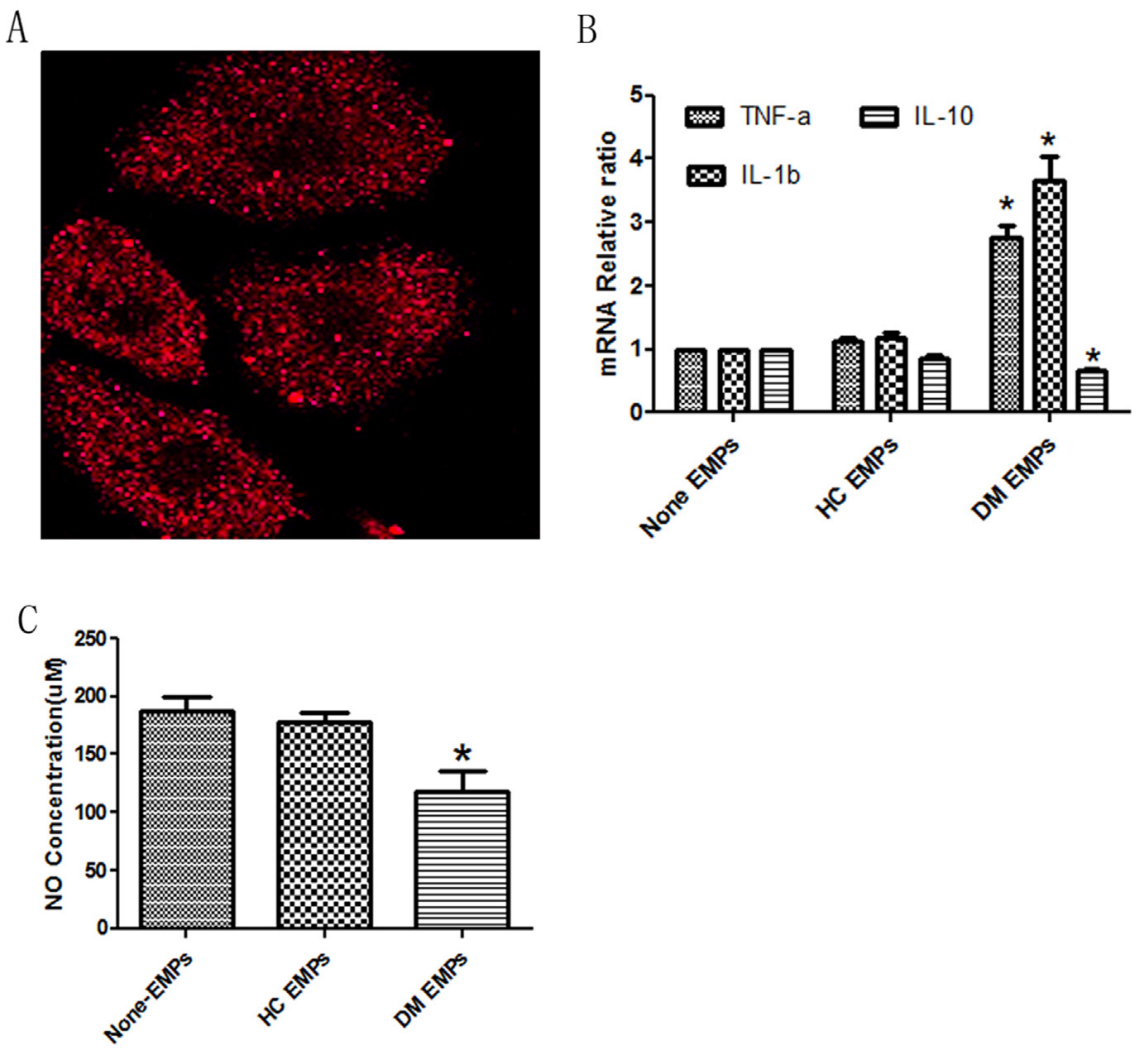
DM patient-derived EMPs induce HUVEC dysfunction **(A)** HUVECs effectively engulf EMPs using confocal. Red, PE-labelled EMPs (600x). **(B)** DM patient-derived EMPs, but not healthy control-derived EMPs, increase the expression of *TNF-a* and *IL-1b* mRNA, whereas they decrease the expression of *IL-10* mRNA. **(C)** DM patient-derived EMPs, but not healthy control-derived EMPs, decrease NO expression in HUVECs. Healthy control, n=6; and DM, n=10. HC EMPs, healthy control-derived endothelial microparticles; and DM patient-derived EMPs, diabetes mellitus patient-derived endothelial microparticles. ^*^ p<0.05. *GAPDH* mRNA serves as an internal control.

As previous reported, the level of EMP at 1x10^5^ EMPs/ml is sufficient to generate effects for target cells [9], therefore we applied EMPs at 1x10^5^ EMPs/ml to cultured HUVECs. As shown in Figure 2A, the plasma EMPs could effectively be engulfed by cultured HUVECs. Then, we measured *IL-1b, TNF-a and IL-10* mRNA levels after EMP stimulation. We found that DM patient-derived EMPs significantly induced *IL-1b* and *TNF-a* mRNA expression, whereas they inhibited *IL-10* mRNA expression compared to healthy volunteer-derived EMPs (Figure 2B), which indicated that DM patient-derived EMPs increased inflammatory release in cultured ECs. Additionally, we also measured NO expression. As shown in Figure 2C, DM patient-derived EMPs decreased NO expression in ECs. Combining these data, we deduced that DM patient-derived EMPs induced EC dysfunction *ex vivo*.

### High glucose levels promoted EMP release *ex vivo*

**Figure 3:**
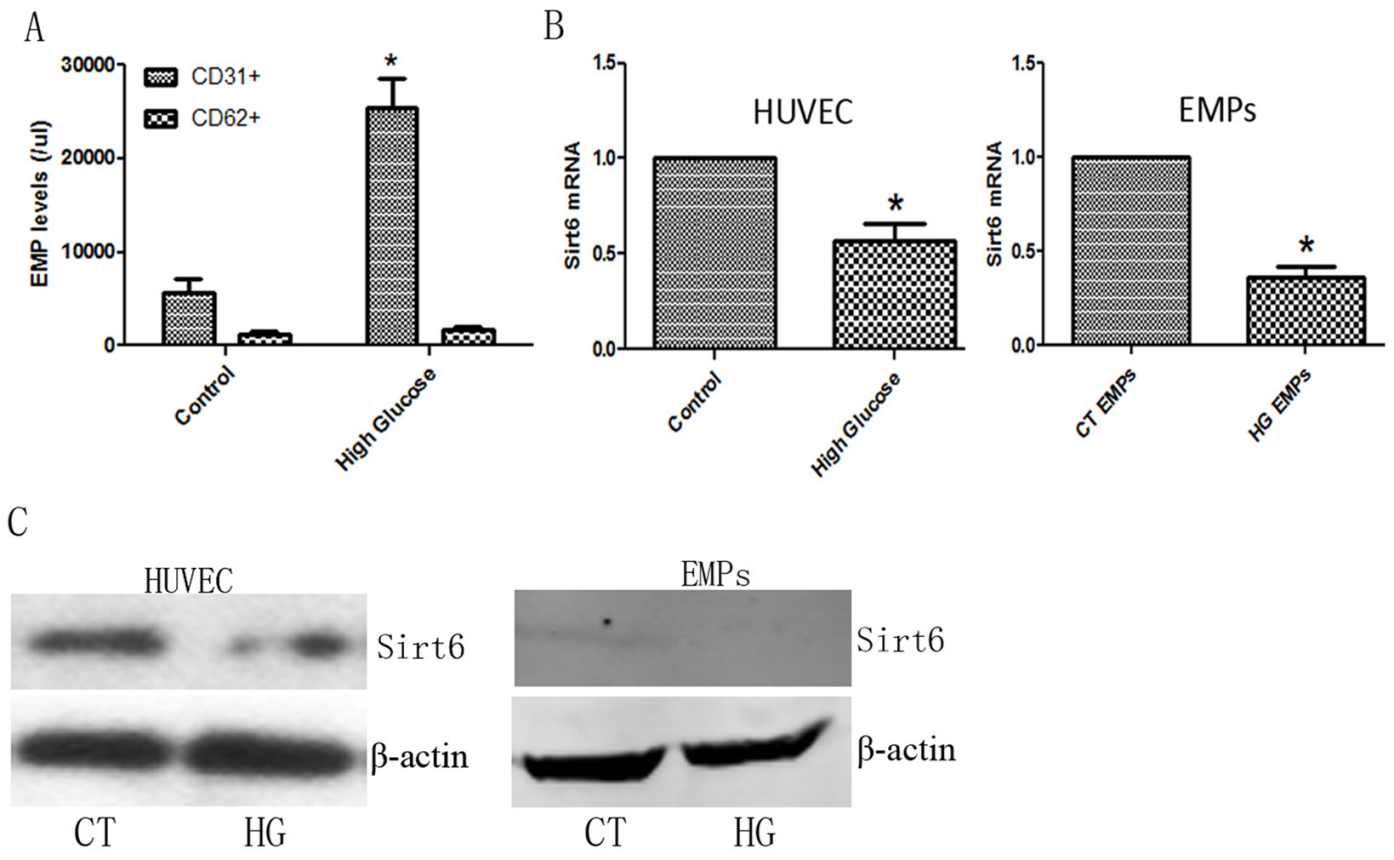
EMP levels and Sirt6 expression in high glucose-cultured HUVECs **(A)** High glucose induces the formation of apoptotic (CD31+) EMPs but not activated (CD62+) EMPs. **(B)** High glucose decreases *Sirt6* mRNA expression in HUVECs and EMPs. **(C)** High glucose decreases Sirt6 protein expression in HUVECs but not EMPs. n=3. EMPs, endothelial microparticles; CT, control; and HG, high glucose. ^*^ p<0.05. *GAPDH* mRNA serves as an internal control.

As we shown in previous document [18], 33 mM glucose had sufficient effect to induce HUVECs dysfunction, therefore, we directly applied 33 mM glucose to cultured HUVECs to verify the relationship between DM and EMP release. As expected, high glucose levels increased CD31+ EMP production (Figure 3A). Additionally, we detected the expression of *Sirt6 mRNA* and protein in EMPs and HUVECs. As with DM patients, high glucose levels inhibited *Sirt6* mRNA and protein expression in EMPs and ECs (Figure 3B, 3C and Supplementary Figure 1B).

### *Sirt6 mRNA*-containing EMPs control EMP-mediated HUVEC dysfunction *ex vivo*

**Figure 4:**
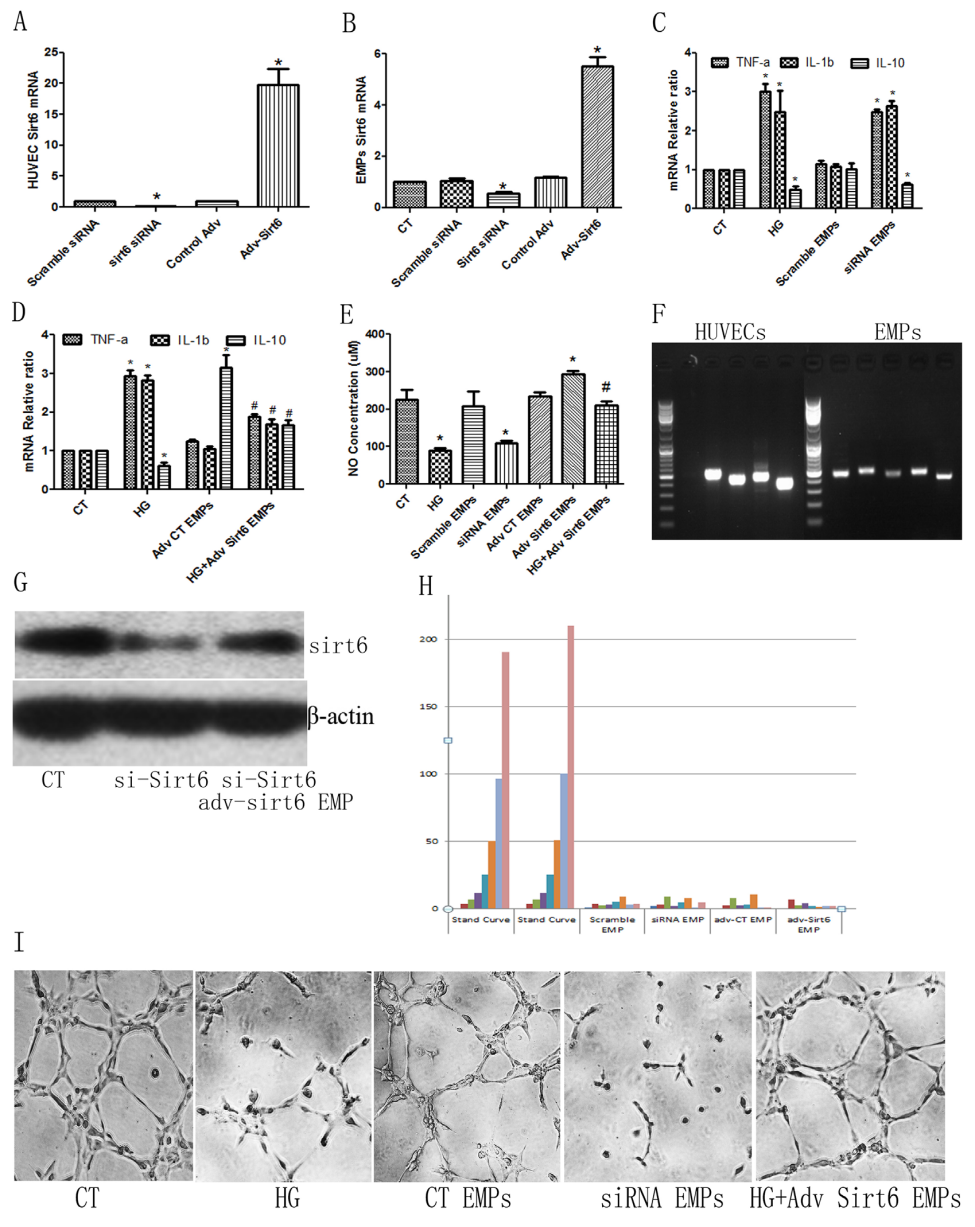
*Sirt6* mRNA plays important roles in high glucose-derived EMP-induced HUVEC dysfunction **(A)** *Sirt6* siRNA or *Adv-Sirt6* alters *Sirt6* mRNA levels in HUVECs. **(B)** *Sirt6* siRNA or *Adv-Sirt6* alters *Sirt6* mRNA levels in EMPs. **(C)** *Sirt6* siRNA-treated EMPs mimic high glucose-induced *IL-10* decreases and *TNF-a* and *IL-1b* increases. **(D)** *Adv-Sirt6*-treated EMPs protect against high glucose-induced *IL-10* decreases and *TNF-a* and *IL-1b* increases. **(E)** *Sirt6* siRNA-treated EMPs mimic high glucose-induced NO decreases, whereas *Adv-Sirt6*-treated EMPs protect against high glucose-induced NO decreases. **(F)** PCR of *Sirt6 mRNA* incorporated in EMPs and HUVEC. **(G)** *Adv-Sirt6* EMPs rescues Sirt6 protein expression in *Sirt6 KD* HUVECs. *si-Sirt6, Sirt6 siRNA*. **(H)** Sirt6 protein in EMPs is undetectable using ELISA. **(I)** High glucose- and *Sirt6 siRNA*-treated EMPs impair HUVEC tube formation, whereas *Adv-Sirt6*-treated EMPs induce tube formation in high glucose-cultured HUVECs. n=3. CT, control; HG, high glucose; EMPs, endothelial microparticles; siRNA EMP, Sirt6 siRNA treated-EMP; Adv Sirt6 EMP, adv-Sirt6 treated-EMP. ^*^ p<0.05 vs CT; ^#^ p<0.05 vs HG. *GAPDH* mRNA serves as an internal control. “CT EMPs” means that EMPs sourced HUVECs did not undergo siRNA- or adv- contamination.

As *Sirt6 mRNA* expression decreased in DM patient- or high glucose-derived EMPs, we suspected that Sirt6 deficiency may be related to EMP-mediated HUVEC dysfunction. First, we applied *Sirt6 siRNA* or *Adv-Sirt6* to generate *Sirt6*-deficient or *Sirt6*-enriched EMPs (Figure 4A, 4B). Then, we added these EMPs to cultured-HUVECs. Interestingly, *Sirt6*-deficient EMPs effectively mimicked high glucose- derived EMPs and mediated EC dysfunction, including increased inflammatory cytokine production and decreased NO production, whereas *Sirt6*-enriched EMPs reversed the high glucose-derived EMP-mediated EC dysfunction (Figure 4C-4E). Angiogenesis is a very important sign of ECs favorable function, therefore we added EMPs to HUVECs. As shown in Figure 4I and Supplementary Figure 1D, high glucose and *Sirt6-mRNA*-deficient EMPs impeded HUVECs tube formation, whereas Adv-*Sirt6*-treated EMPs induced tube formation in high glucose-cultured HUVECs.

To prove that mRNAs incorporated in the EMPs are mature and complete *Sirt6 mRNA*, we then measured the EMPs *Sirt6 mRNA* sequence using RNAseq. As shown in Figure 4F and Supplementary Figure 2, the *Sirt6 mRNAs* incorporated in the EMPs are mature. To further confer the *Sirt6 mRNA* incorporated in the EMPs are functional, we added *Sirt6 mRNA*-enriched EMPs to *Sirt6* knock down (KD) HUVECs, which induced by *Sirt6* siRNA. As shown in Figure 4G and Supplementary 1C, *Sirt6 mRNA*-enriched EMPs partially rescued Sirt6 protein expression in *Sirt6 KD* HUVECs. Additionally, to exclude whether these effects were caused by free Sirt6 protein, which is centrifuged together with EMPs, or Sirt6 proteins which is contained in EMPs, we lysed the EMPs precipitate directly and then measured using ELISA. Interestingly, the Sirt6 levels were undetectable (Figure 4H).

**Figure 5:**
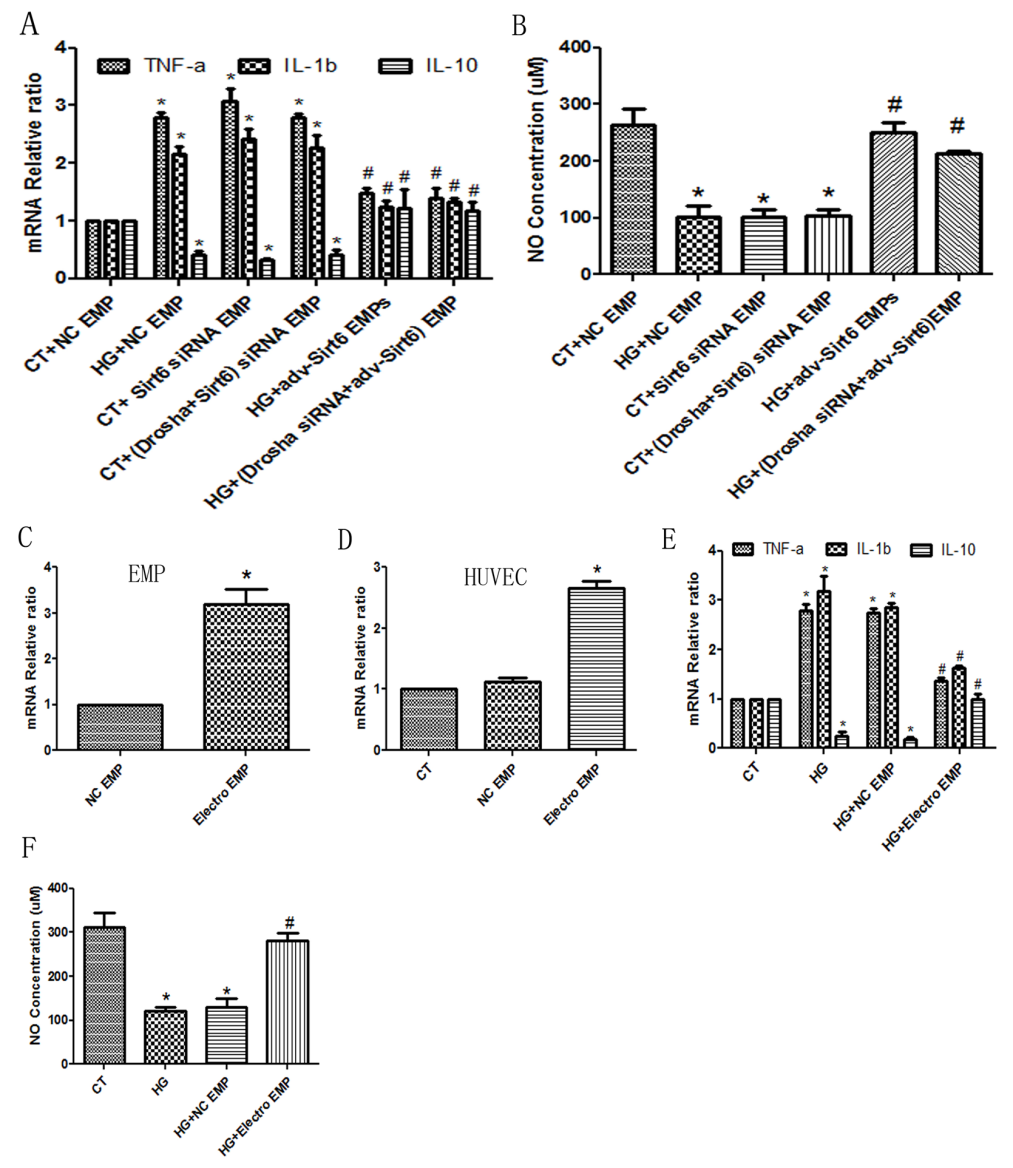
*Sirt6 mRNA* incorporated in EMPs is a key factor for HUVEC function *Drosha siRNA* was used to generate miRs-deficient EMPs. **(A)** *Sirt6* siRNA-treated EMPs mimic high glucose-induced *IL-10* decreases and *TNF-a* and *IL-1b* increases whether *Drosha siRNA* presence or absence in EMP formation. Whereas *Adv-Sirt6*-treated EMPs protect against high glucose-induced *IL-10* decreases and *TNF-a* and *IL-1b* increases whether *Drosha siRNA* presence or absence in EMP formation. **(B)** *Sirt6* siRNA-treated EMPs mimic high glucose-induced NO decreases, whereas *Adv-Sirt6*-treated EMPs protect against high glucose-induced NO decreases whether *Drosha siRNA* presence or absence in EMP formation. **(C)** Electroporation transfers *Sirt6 mRNA* into EMPs. **(D)** Eletroporation-EMP effectively transfers *Sirt6 mRNA* into target HUVECs. **(E)** Electroporation-EMPs protect against high glucose-induced *IL-10* decreases and *TNF-a* and *IL-1b* increases. **(F)** Electroporation-EMPs protect against high glucose-induced NO decreases. n=3. EMPs, endothelial microparticles; CT, control; and HG, high glucose; Electro, electroporation. ^*^ p<0.05 vs CT; ^#^ p<0.05 vs HG. *GAPDH* mRNA serves as an internal control.

Undisputedly, the primary nucleotides species found and the most popular research focused in EMPs are microRNAs (miRs) [5], therefore we first using *Drosha siRNA* in cultured-HUVECs to impede miRs package, then added *Sirt6 siRNA* or *Adv-Sirt6* into these cells to generate EMPs. After careful collection, these miRs-deficient EMPs were added to cultured-HUVECs. Interestingly, *Sirt6 mRNA*, which is incorporated in the EMPs, rescued the ECs dysfunction caused by high glucose, including decreased inflammatory cytokine production and increased NO production, still preserved (Figure 5A, 5B). Additionally, we also packaged *Sirt6 mRNA* into EMPs using mRNA electroporation method. As shown in Figure 5C-5D, *Sirt6 mRNA* can effectively transferred into EMPs and then conveyed to target HUVECs. We also observed the inflammatory factors release and NO secretion. As shown in Figure 5E, EMPs packaged *Sirt6 mRNA* inhibited *IL-1b* and *TNF-a* release but induced *IL-10* release. Also the NO expression is increase following Sirt6-enriched EMPs treatment (Figure 5F).

### eNOS expression differed in HUVECs following exposure to EMPs

**Figure 6:**
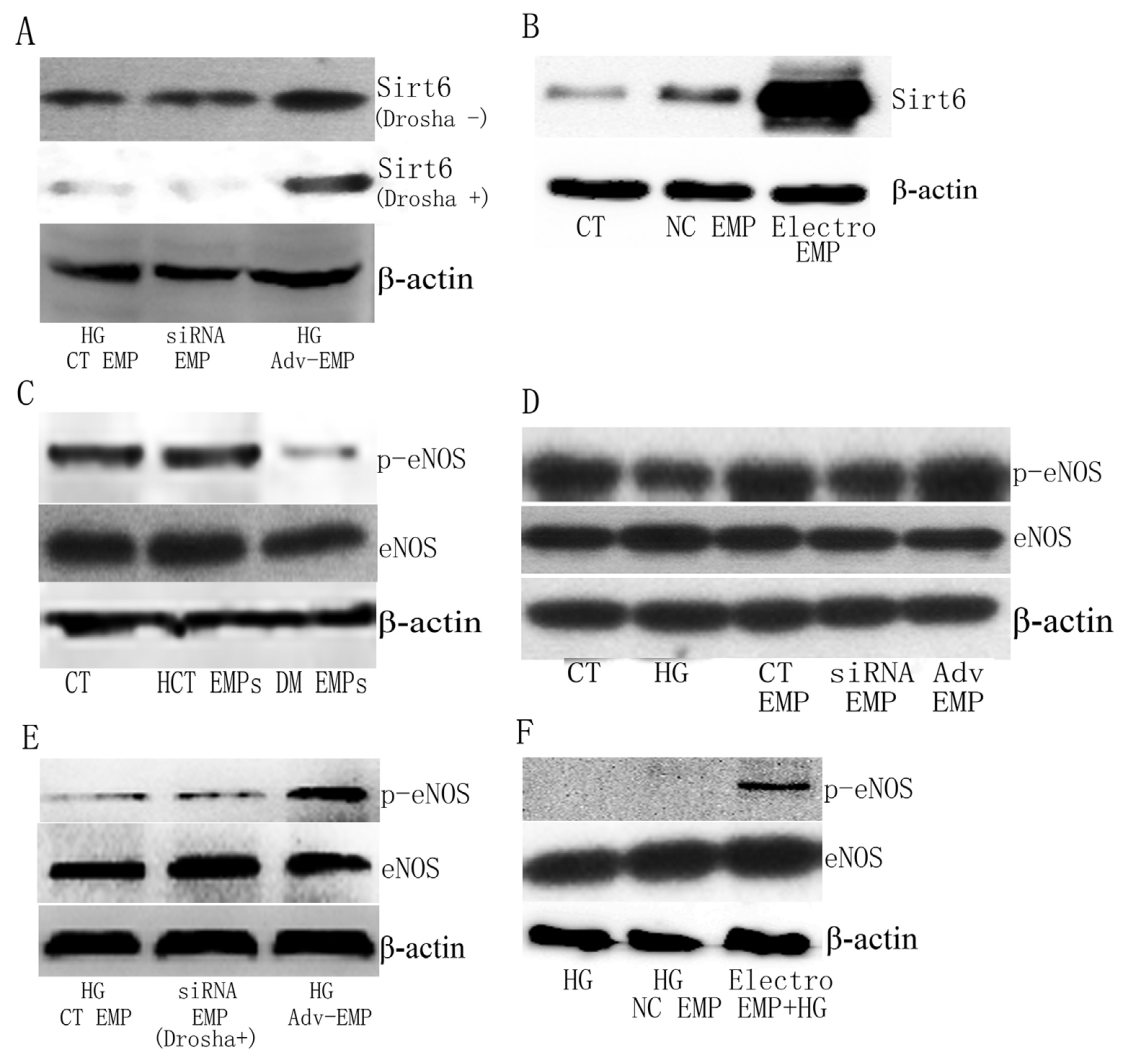
Sirt6 protein and phosphorylation of eNOS is different in HUVECs following exposure to EMPs **(A)** *Sirt6 mRNA*-incorporated EMPs, in spite of miRs-enrichment (*Drosha* -) or miRs-deficient (*Drosha* +), alter Sirt6 protein expression in HUVECs. **(B)** Electroporation-EMPs alter Sirt6 protein expression in HUVEC. **(C)** DM patient-derived EMPs, but not healthy control-derived EMPs, cause a decrease in p-eNOS in HUVECs. **(D)** High glucose- and *Sirt6-siRNA*-treated EMPs decease p-eNOS levels, whereas Adv-Sirt6-treated EMPs increase p-eNOS levels in HUVECs following high glucose administration. **(E)** *Sirt6 mRNA*-incorporated EMPs, which are miRs-deficient, induce p-eNOS increase in HUVECs. **(F)** *Sirt6 mRNA* electroporation-EMPs increases p-eNOS levels in HUVECs following high glucose administration. n=3. CT, control; HCT, healthy control; HG, high glucose; DM, diabetes mellitus; EMPs, endothelial microparticles; Electro, electroporation.

To verify that *Sirt6 mRNA* did not degrade after transfection, we evaluated Sirt6 protein expression in HUVECs following EMP administration in spite of miRs elimination (using *Drosha siRNA*) or mRNA electroporation (Figure 6A-6B and Supplementary Figure 1E-1F). Then, we evaluated the activation of eNOS, which is a key mediator in the Sirt6 pathway. Immunoblotting analysis revealed decreased phosphorylation of eNOS when HUVECs were exposed to DM patient-derived EMPs compared with exposure to healthy volunteer-derived EMPs, suggesting that eNOS is inactive following stimulation by DM patient-derived EMPs (Figure 6C and Supplementary Figure 1G). The effect of *Sirt6 mRNA*-containing EMPs on eNOS was further confirmed. The relative amount of p-eNOS following *Sirt6 mRNA*-enriched EMP exposure was significantly increased, whereas *Sirt6 mRNA*-deficient EMPs mediated a significant downregulation of eNOS phosphorylation (Figure 6D-6F and Supplementary Figure 1H-1J). These data indicated that eNOS phosphorylation may participate in EMP-mediated EC dysfunction in DM.

### *Sirt6 mRNA*-containing EMPs improved ECs function is independent of *Sirt1-*dependent manner

**Figure 7:**
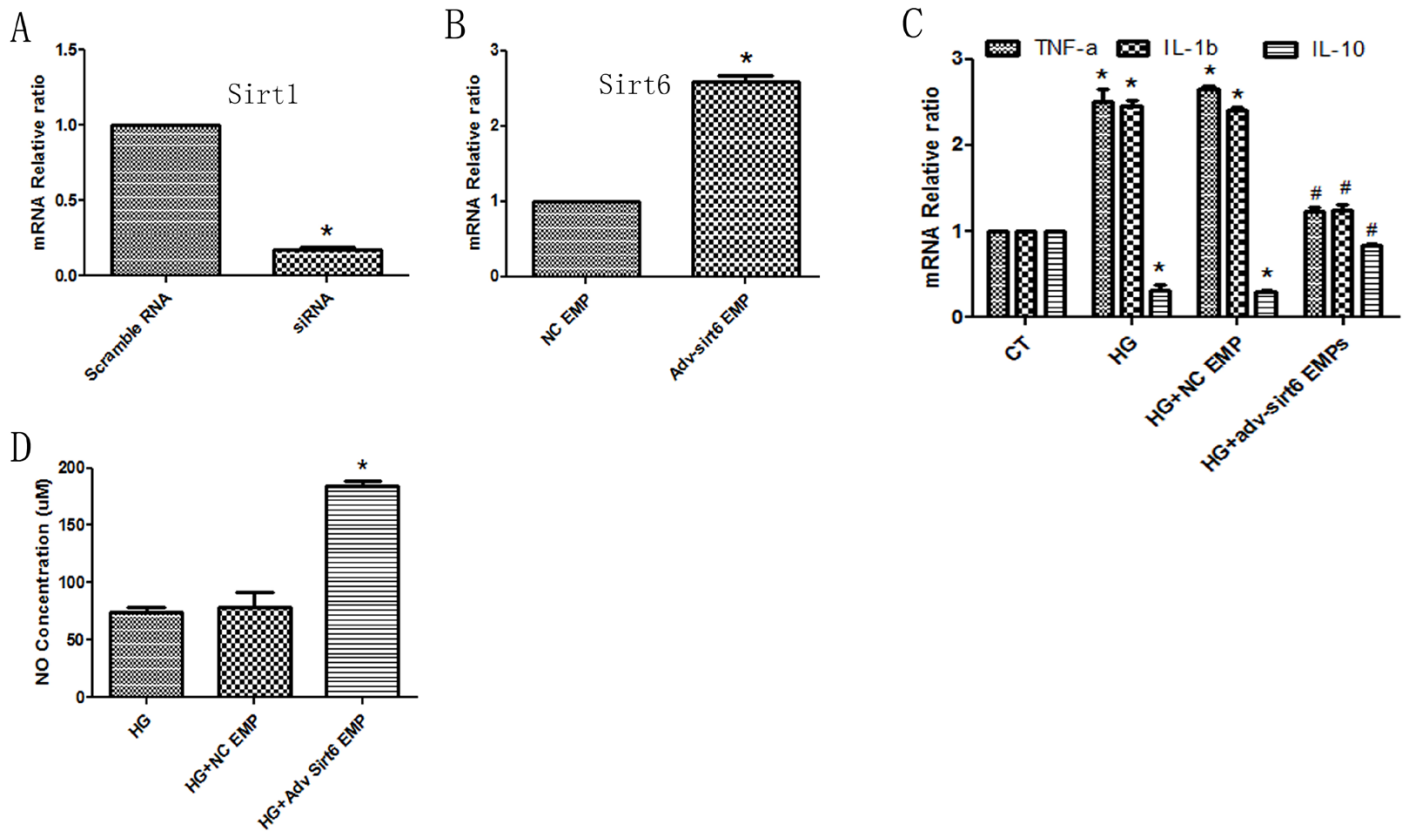
*Sirt6 mRNA*-mediated protective effects are independent of *Sirt1* **(A)** *Sirt1 siRNA* abrogates *Sirt1 mRNA* expression in HUVECs. **(B)** *Adv-Sirt6*-treated EMPs increase *Sirt6 mRNA* levels in *Sirt1 KD* HUVECs. **(C)** *Adv-Sirt6*-treated EMPs protect against high glucose-induced *IL-10* decreases and *TNF-a* and *IL-1b* increases in *Sirt1 KD* HUVECs. **(D)** *Adv-Sirt6*-treated EMPs protect against high glucose-induced NO decreases in *Sirt1 KD* HUVECs. CT, control; HG, high glucose; EMPs, endothelial microparticles. ^*^ p<0.05 vs CT; ^#^ p<0.05 vs HG. *GAPDH* mRNA serves as an internal control.

Previous documents proven that Sirt1-dependent mechanism was wide agreement in DM-associated endothelial dysfunction [19], therefore we added *Sirt6 mRNA*- containing EMPs into *Sirt1* deficient HUVECs (Figure 7A, 7B). We found that the *IL-1b* and *TNFa* mRNAs were decrease, whereas *IL-10* mRNA was increase (Figure 7C). Additionally, NO production was induced following Sirt6 mRNA-enriched EMPs treatment (Figure 7D). These data indicated that *Sirt6 mRNA*-containing EMPs improved ECs function is independent of, at least partially, *Sirt1-*dependent manner.

## DISCUSSION

The present study demonstrated that DM-derived EMPs activate inflammatory cytokines and decrease the production of NO in cultured HUVECs, thereby facilitating EC damage. Notably, EMPs carrying *Sirt6 mRNA* are critically involved in the process, and regulation of EMPs containing Sirt6 could effectively affect EMP-mediated inflammatory cytokine activation, NO decrease and impaired tube formation. In addition, the phosphorylation of eNOS was revealed to be associated with the stimulation of ECs by EMPs.

The potential role of endothelial dysfunction in cardiovascular complications has recently been investigated using cultured ECs and experimental models of DM. Indeed, endothelial dysfunction manifests as endothelial erosion, apoptosis, inflammation and premature senescence with pronounced EMP release [10, 18]. Therefore, the possibility that dysfunctional endothelial cells are a major source of deleterious EMPs in DM patients was evaluated. The present findings further confirmed these previous hypotheses, showing that both EMPs released from activated endothelial cells and apoptotic endothelial cells are upregulated in DM patients, whereas the appearance of a pronounced CD31+/CD42-, apoptotic EMP profile, but not a CD62+/CD42-, activated EMP profile, is observed in high glucose-exposed HUVECs. Notably, the prevalence of CD31+/CD42- EMPs is much higher in DM patients than CD62+/CD42- EMPs. In addition to representing a surrogate diagnostic and therapeutic marker of endothelial dysfunction, EMPs might also contribute to aggravating the function of circulating ECs, as EMPs from patients with DM exerted prothrombotic, proinflammatory, disseminated intravascular coagulopathic, microvascular injury and immunosuppressive effects on circulating ECs [20]. They also blunted endothelium-dependent relaxation in arterial rings [21, 22] and promoted premature endothelial senescence and impaired tube formation in cultured ECs [21]. At present, the uptake of EMPs from DM patients or high-glucose incubated HUVECs increases proinflammatory cytokine release, reduces NO release and impairs tube formation, whereas a similar concentration of EMPs from healthy volunteers are inactive on ECs. Altogether, these findings support the concept that DM patient-derived EMPs carry a pro-damage message to neighboring ECs.

In addition to classical short-distance intercellular communication via signaling molecules and long-distance communication via hormones, extracellular vesicles, such as microparticles and exosomes, have been proposed to be essential for information exchange that affects the physiology of neighboring and remote recipient cells [23, 24]. Mostly evidence suggests that the information transferred and then elicited pathophysiological effects on the target cells by microparticles and exosomes are mediated by microRNA or protein-dependent mechanisms [25, 26]. Recently, researchers found that mRNA incorporated into MPs also played key roles in tumor biology and predicted prognostic risk stratification [27]. However, there is no doubt that the cargo that is incorporated into the EMPs is dependent on the stimulus received by the parent ECs [28]. miR-126, membrane-associated proteins and glycoproteins that are incorporated into the EMPs are accepted as the major mechanism by which information is transferred to neighboring and remote recipient cells [22, 29]. Sirt6 is a sirtuin family member that participates in the control of a wide range of pathophysiological processes, including glucose homeostasis, cardiovascular diseases, cancer and longevity [30]. The relationship between Sirt6 and diabetes is complex, although Sociali G. reported that pharmacological Sirt6 inhibition improved glucose tolerance as well as reduced triglycerides and cholesterol levels in a T2DM mouse model [31]; most models have confirmed the hypothesis that Sirt6 serves as a surrogate diagnostic and therapeutic marker of DM with or without cardiovascular complications. Sirt6 expression decreases in diabetic atherosclerotic plaque and endothelium [32], whereas overexpression of Sirt6 protects against endothelial senescence [17], inhibits macrophage-derived foam cell formation [33], represses aging [34], delays pancreatic β-cell dysfunction and apoptosis [35], and enhances insulin sensitivity [36]. Downregulation of Sirt6 accelerates ROS- or high glucose-induced endothelial senescence [17, 37] and leads to obesity and insulin resistance [38]. At present, we also determined that serum Sirt6 levels are much lower in diabetic patients than in healthy controls. However, there is limited information available regarding Sirt6 expression in EMPs from DM patients. Interestingly, the expression of *Sirt6 mRNA*, but not protein, in the EMPs from DM patients and high glucose-incubated HUVECs is consistently decreased; additionally, *Sirt6 mRNA* and protein deficiency has also been confirmed in HUVECs following high glucose treatment in our study, suggesting that Sirt6 might have significant effects on the pathogenesis of diabetic complications. Additionally, altering *Sirt6 mRNA* concentrations in EMPs using *Adv-Sirt6*, *Sirt6 siRNA* or direct mRNA-electroporation can consistently direct EMP-mediated effects on target HUVECs, including an inflammatory phenotype, NO production and tube formation. These effects also exist using miRs-deficient EMPs. Combining these data, we may deduce that *Sirt6 mRNA*-incorporated EMPs may mediate an important mechanism in the transfer of information to neighboring and remote ECs in diabetes.

It is well known that eNOS-derived NO formation plays a very important role in maintaining endothelial functional integrity. Indeed, both senescent and apoptotic ECs are observed with a pronounced downregulation of eNOS expression and NO formation [18, 39]. Recently, researchers have observed that EMPs from CAD or CHF patients inhibit angiogenesis and induce inflammation in ECs via disturbed eNOS phosphorylation and NO formation [40, 41]. The present findings further extend these previous reports by showing that blunted eNOS phosphorylation and NO formation are observed in HUVECs following exposure to EMPs from DM patients or high glucose-incubated ECs, suggesting that the eNOS pathway is very important for the transfer of information via EMPs in DM. Whether the EMP-mediated decrease of eNOS phosphorylation and NO formation is related to Sirt6 is still unclear. Although Liu R reported that the downregulation of Sirt6 decreased eNOS phosphorylation and played key roles in oxidative stress-induced EC senescence [37], the results contrasted with those from Freitas M who showed that Sirt3 and Sirt7, but not Sirt6, had a close relationship with eNOS phosphorylation and NO formation in aged patients [32]. At present, our findings further extend these previous reports by showing that blunted eNOS phosphorylation and NO formation can be observed in target ECs following Sirt6-deficient EMP exposure, whereas *Sirt6 mRNA*-overexpressing EMPs cause significant eNOS phosphorylation and NO formation. Thus, it may be possible that Sirt6 deficiency in DM patient-derived EMPs induces endothelial dysfunction via the eNOS pathway.

There is a limitation in this study that should be noted. Although we have shown that *Sirt6 mRNA*-incorporated EMPs are very important for the maintenance of neighboring and remote EC function, the mechanism underlying the effects of Sirt6 on the eNOS pathway has not been completely elucidated. Additionally, direct eNOS regulation in target HUVECs was not shown in this study. Therefore, additional *in vivo* and *ex vivo* studies are needed to verify the abovementioned mechanisms.

In conclusion, diabetes leads to increased EMP release and decreased serum Sirt6 levels. *Sirt6 mRNA*-incorporated EMPs are decreased in DM patients and high glucose-incubated HUVECs. EMPs containing *Sirt6 mRNA* inhibits inflammatory cytokine release and induces tube formation, whereas deficiency of *Sirt6 mRNA* in EMPs amplifies inflammation and impairs tube formation. Moreover, eNOS phosphorylation and NO formation may be involved in this progress. Many questions must be answered regarding the molecular network underlying these responses. However, the possibility that the *Sirt6 mRNA*-incorporated EMPs have an essential effect on neighboring and remote ECs in DM is very exciting.

## MATERIALS AND METHODS

### Reagents

Glucose and TRIzol were purchased from Sigma (St Louis, Missouri, USA). Monoclonal antibodies against CD62-PE, CD31-PE, CD42-FITC and IgG-PE were obtained from BD (Shanghai, China), and the monoclonal rabbit antibodies against Sirt6, p-eNOS, eNOS and β-actin were obtained from Cell Signaling (Denver, Colorado, USA). The fetal bovine serum (FBS), DMEM culture medium with or without glucose, Lipofectamine® 2000 Transfection Reagent and ECMatirm™ solution were purchased from Invitrogen (Carlsbad, CA, USA). The cDNA Synthesis Kit and Premix Ex Taq SYBR Green PCR Kit were purchased from Takara (Shiga, Japan). The *adv-Sirt6, Sirt6 siRNA, CMV-Sirt6 shRNA, Sirt1 siRNA* and *Drosha siRNA* were obtained from HanBio. (Shanghai, China). Other unmentioned reagents were purchased from Shenggong Bio. (Shanghai, China).

### Patients and blood collection

Informed consent was obtained from all participants in accordance with the guidelines of the Human Subjects Committee of the Medical Ethics Commission of the Tongji Hospital Affiliated with Tongji University (Shanghai, China). Between August 2014 to March 2015, 10 diabetes patients without hypertension, smoking, chronic kidney disease, peripheral atherosclerosis, inflammation, malignant tumors, severe hepatic dysfunction or coronary atherosclerosis who were diagnosed by coronary angiogram at our inpatient department were screened for inclusion in the study. Six age-matched healthy volunteers were also included in our present study. All DM patients were treated with either oral antidiabetic drugs or subcutaneous insulin administration.

Venous blood was obtained from the cubital vein under sterile conditions and then buffered with sodium citrate (for EMP analysis) or with anticoagulant sodium (for Sirt6 quantification). Blood was centrifuged at 1,500 g for 10 min, and then, the supernatant was centrifuged at 13,000 g for 20 min to obtain MPs, which were subsequently resuspended in PBS. The blood was coagulated for 45 min, and then, the serum was collected to measure Sirt6 levels.

### Cell culture

The human umbilical vein endothelial cell (HUVEC) line and endothelial cell media (ECM) were obtained from ScienCell (Carlsbad, California, USA). The HUVECs were grown in ECM (glucose: 5.5 mM), starved by culturing them without FBS for 12 h and then exposed to glucose at concentrations of 0 mM for 24 h or 33 mM for 72 h to generate EMPs. Additionally, *Adv-Sirt6* or *Sirt6 siRNA* was used to alter EMP components.

### Adv-Sirt6 transfection

*Adv-control* or *Adv-Sirt6* was directly added to cultured cells and incubated for 24 h.

### siRNA transfection

Cells were transfected with either *scramble siRNA, Sirt6 siRNA, Sirt1 siRNA* or *Drosha siRNA* using Lipofectamine® 2000 Transfection Reagent following the manufacturer’s procedure. The *siRNA* sequence is shown on Table 1.

**Table 1:** Oligonucleotide sequences of the primers and siRNAs

Genes	Oligonucleotide sequences	Protocols
Primers		
*TNF-a*	Forward: 5′-CCGTCTCCTACCAGACCAAGG-3′	
	Reverse: 5′-CTGGAAGACCCCTCCCAGATAG-3′	
*IL-1b*	Forward: 5′-CTGATGGCCCTAAACAGATGAAG-3′	
	Reverse: 5′-GGTCGGAGATTCGTAGCAGCTGGAT-3′	
*IL-10*	Forward: 5′-CATGCTGCTGGGCCTGAA-3′	
	Reverse: 5′-CGTCTCCTTGATCTGCTTGATG-3′	
*Sirt6*	Forward: 5′-GCCCCGCTTCCGGCGGAAG-3′	
	Reverse: 5′-ATTGTTTTTATTGCATTGAGGAC-3′	
*Drosha*	Forward: 5′-TCCCTGCTGCCCAAGATTTC-3′	stage1: 95°C, 30 sec
	Reverse: 5′-CAGGGCTTTGCTGCACCTTA-3′	stage2: 95°C, 5 sec
*Sirt1*	Forward: 5′-TGTGGTAGAGCTTGCATTGATCTT-3′	55°C, 30 sec
	Reverse: 5′-GGCCTGTTGCTCTCCTCATT-3′	72°C, 35 sec
GAPDH	Forward: 5′-AAGGTGAAGGTCGGAGTCA-3′	40 cycles
	Reverse: 5′-GGAAGATGGTGATGGGATTT-3′	
siRNA		
Sirt6	Sense: 5′-CCCUGGUCUCCAGCUUAAATT-3′	
	Anti-Sense: 5′-UUUAAGCUGGAGACCAGGGTT-3′	
Sirt1	Sense: 5′-GATTATTGCCGGAAACAA-3′	
	Anti-Sense: 5′-TCCTTTCAGAACCACCAAA-3′	
Drosha	Sense: 5′-GGATTAGCAACCTATCGGA-3′	
	Anti-Sense: 5′-AAGGACCAAGTATTCAGCA-3′	
GAPDH:	Sense: 5′-GUAUGACAACAGCCUCAAGTT-3′	
	Anti-Sense: 5′-CUUGAGGCUGUUGUCAUACTT-3′	

### EMP collection from HUVECs

After incubation with *Adv-Sirt6, Sirt6 siRNA* or *Drosha siRNA* for 24 h, the supernatant was removed, and the cells were carefully washed with PBS 3 times for 5 min. Then, the cells were exposed to glucose at concentrations of 0 mM for 24 h or 33 mM for 72 h. The medium was collected and centrifuged at 1000 rpm for 5 min, and then, the supernatant was centrifuged at 13000 g for 20 min to obtain MP, which were resuspended in PBS.

### Quantitative determination of the plasma EMP levels or cultured HUVEC EMP levels

As previous description, CD31 and CD62 were biomarkers of endothelial cells, whereas CD42 was biomarker of platelets, therefore, CD62+/CD42- or CD31+/CD42- were considered to be EMPs in plasma. Usually, documents reported that CD62+ EMPs released from activated endothelial cells, whereas CD31+ released from apoptotic endothelial cells [2]. CD31+/CD42- or CD62+/CD42- MPs levels were measured freshly with flow cytometry by using CD42-FITC and CD31-PE/CD62-PE.

### Real-time reverse transcription PCR (RT-PCR)

Total RNA from HUVECs or MPs (obtained using the TRIzol procedure) was converted to cDNA using Takara reverse transcriptase. RT-PCR was performed using the samples and SYBR Premix regent on an ABI 7500. The primers (Shenggong Bio., China) are displayed on Table 1.

### RNA-seq

Total RNA from HUVECs or MPs was tested for quality and treated with DNase and RiboZero Kits (Illumina, Singapore) to remove DNA and rRNA. RNA-seq was performed by Shenggong Bio., China.

### mRNA electroporation

After purified of mRNA using Oligotex mRNA Mini Kit (Qiagen, Germany), the *Sirt6 mRNA* was executed of electroporation into EMPs was performed using GenePulser XcellTM electroporation system (BioRad, USA) as previously described [42]. Briefly, 1x10^5^ EMPs and 4 ug mRNA were mixed in 200 ul of electroporation buffer and electroporated at 350 V and 150 uF in a 10 msec. After incubated at 37°C for 30 min, the EMPs were transferred to cultured cells.

### Western blotting

Equal amounts of total protein from HUVEC or MPs (obtained using cell total protein lysis buffer, Beyotime, Haimen, China) were subjected to 8-10% SDS-PAGE, transferred to PVDF membranes and then blocked using 5% non-fat milk. After incubation with primary antibodies at 4 °C overnight, HRP-conjugated secondary antibodies were applied at room temperature for 1 h. Immunoblots were visualized using enhanced chemiluminescence reagents (Bio-Max, Israel).

### Enzyme-linked immunosorbent assay of Sirt6 production in blood and EMPs

Sirt6 production was detected in blood or EMPs using human ELISA kits according to the manufacturer’s instructions.

### HUVEC tube formation assay in ECMatirm™ solution

To assess the vasculogenic effects of EMPs on endothelial cells *ex vivo*, an ECMatirm™ solution tube formation assay was performed according to the manufacturer’s instructions.

### NO assay

NO levels in culture supernatants were measured using the nitrite method (Jiancheng, Nanjing, China). Briefly, the reaction substrate was mixed with supernatants, vortexed, incubated and then centrifuged at 4,500 rpm for 15 min. The supernatant was then incubated with the chromogenic reagent for 15 min. Finally, the output was detected at an optical absorbance of 540 nm.

### Statistical analyses

Significant differences were determined by one-way ANOVA followed by Tukey’s HSD test using SPSS14.0 software, and all data are presented as the mean ± SEM. A value of p<0.05 was considered statistically significant.

## SUPPLEMENTARY MATERIALS FIGURES



## Abbreviations

DM: diabetes mellitus
EMPs: endothelial microparticles
ECs: endothelial cells
Sirt6: sirtuin 6
Sirt1: sirtuin 1
MPs: microparticles
HUVEC: the human umbilical vein endothelial cells
CAD: coronary artery disease
CHF: chronic heart failure
