# Supplementary material for: Sirt6 mRNA-incorporated endothelial microparticles (EMPs) attenuates DM patient-derived EMP-induced endothelial dysfunction
Source: Oncotarget. 2017 Dec 15;8(69):114300–13. doi: 10.18632/oncotarget.23259 (PMC5768405; doi:10.18632/oncotarget.23259)
Supplement: Supplementary file 1 [file oncotarget-08-114300-s001.pdf]

Sirt6 mRNA-incorporated endothelial microparticles (EMPs) attenuates DM patient-derived EMP-induced endothelial dysfunction

SUPPLEMENTARY MATERIALS

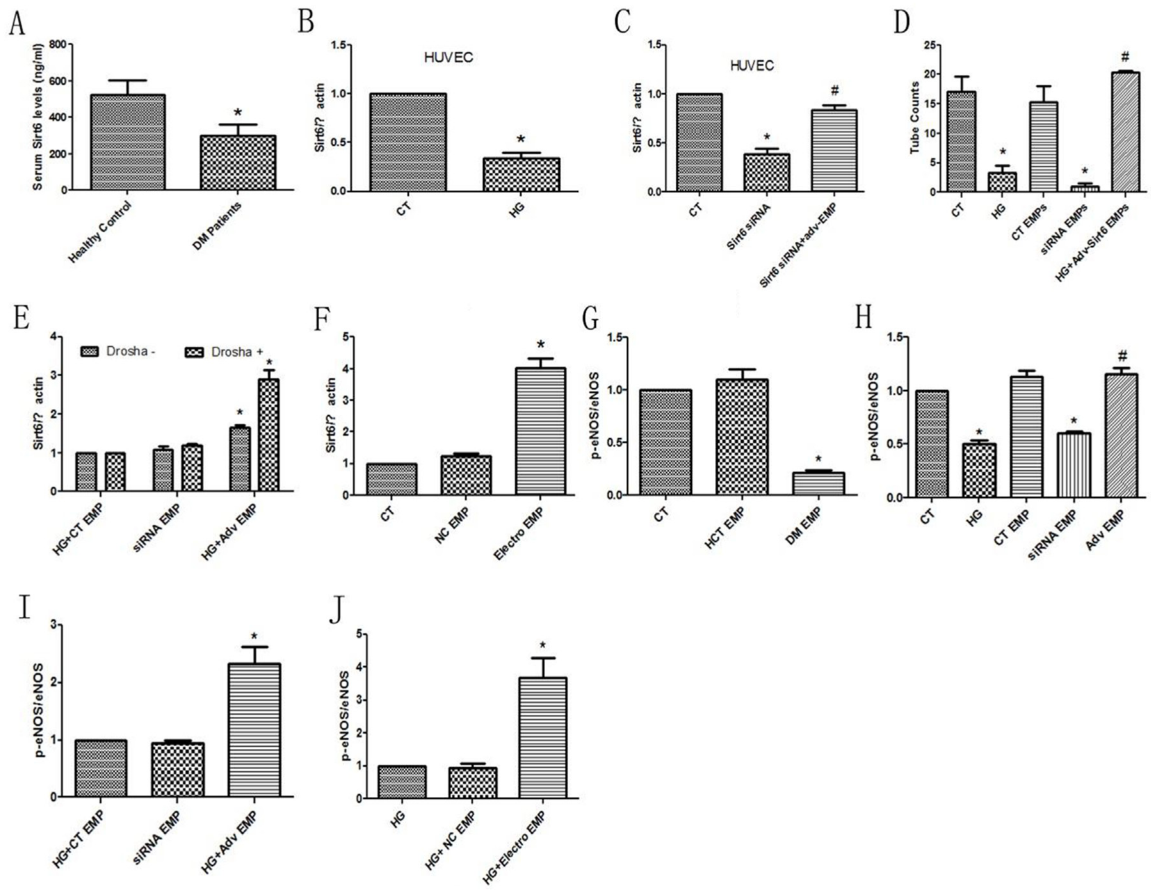

Supplementary Figure 1: Quantitative analysis of tube counts and western blot. (A) Quantitative analysis of Fig. 1D. * p<0.05 vs healthy control. **(B)** Quantitative analysis of Figure 3C in HUVEC. * p<0.05 vs CT. **(C)** Quantitative analysis of Figure 4G in HUVEC. * p<0.05 vs CT; # p<0.05 vs *Sirt6* siRNA. **(D)** Quantitative analysis of Fig. 4I. * p<0.05 vs CT; # p<0.05 vs HG. **(E)** Quantitative analysis of Fig. 6A. * p<0.05 vs HG+CT EMP. **(F)** Quantitative analysis of Fig. 6B. * p<0.05 vs CT. **(G)** Quantitative analysis of Fig. 6C. * p<0.05 vs CT. **(H)** Quantitative analysis of Fig. 6D. * p<0.05 vs CT; # p<0.05 vs HG. **(I)** Quantitative analysis of Fig. 6E. * p<0.05 vs HG+CT EMP. **(J)** Quantitative analysis of Fig. 6F. * p<0.05 vs HG.

HUVEC

GGCCTCAACAAGGGAAACTTTATTGTTCCCGTGGGGCAGTCGAGGATGTCGGTGAATTACGCG
GCGGGGCTGTCGCCGTACGCGGACAAGGGCAAGTGCGGCCTCCCGGAGATCTTCGACCCCCC
GGAGGAGCTGGAGCGGAAGGTGTGGGAACTGGCGAGGCTGGTCTGGCAGTCTTCCAATGTGG
TGTTCCACACGGGTGCCGGCATCAGCACTGCCTCTGGCATCCCCGACTTCAGGGGTCCCCACG
GAGTCTGGACCATGGAGGAGCGAGGTCTGGCCCCCAAGTTCGACACCACCTTTGAGAGCGCG
CGGCCCACGCAGACCCACATGGCGCTGGTGCAGCTGGAGCGCGTGGGCCTCCTCCGCTTCCT
GGTCAGCCAGAACGTGGACGGGCTCCATGTGCGCTCAGGCTTCCCCAGGGACAAACTGGCAG
AGCTCCACGGGAACATGTTTGTGGAAGAATGTGCCAAGTGTAAGACGCAGTACGTCCGAGAC
ACAGTCGTGGGCACCATGGGCCTGAAGGCCACGGGCCGGCTCTGCACCGTGGCTAAGGCAAG
GGGGCTGCGAGCCTGCAGGGGAGAGCTGAGGGACACCATCCTAGACTGGGAGGACTCCCTGC
CCGACCGGGACCTGGCACTCGCCGATGAGGCCAGCAGGAACGCCGACCTGTCCATCACGCTG
GGTACATCGCTGCAGATCCGGCCCAGCGGGAACCTGCCGCTGGCTACCAAGCGCCGGGGAGG
CCGCCTGGTCATCGTCAACCTGCAGCCCACCAAGCACGACCGCCATGCTGACCTCCGCATCCA
TGGCTACGTTGACGAGGTCATGACCCGGCTCATGAAGCACCTGGGGCTGGAGATCCCCGCCTG
GGACGGCCCCCGTGTGCTGGAGAGGGCGCTGCCACCCCTGCCCCGCCCGCCCACCCCCAAGC
TGGAGCCCAAGGAGGAATCTCCCACCCGGATCAACGGCTCTATCCCCGCCGGCCCCAAGCAG
GAGCCCTGCGCCCAGCACAACGGCTCAGAGCCCGCCAGCCCCAAACGGGAGCGGCCCACCA
GCCCTGCCCCCCACAGACCCCCCAAAAGGGTGAAGGCCAAGGCGGTCCCCAGCTGACCAGG
GTGCTTGGGGAGGGTGGGGCTTTTTGTAGAAACTGTGGATTCTTTTTCTCTCGTGGTCTCACTT
TGTTACTTGTTTCTGTCCCCGGGAGCCTCAGGGCTCTGAGAGCTGTGCTCCAGGCCAGGGGTT
ACACCTGCCCTCCGTGGTCCCTCCCTGGGCTCCAGGGGCCTCTGGTGCGGTTCCGGGAAGAA
GCCACACCCCAGAGGTGACAGCTGAGCCCCTGCCACACCCCAGCCTCTGACTTGCTGTGTTGT
CCAGAGGTGAGGCTGGGCCCTCCCTGGTCTCCAGCTTAAACAGGAGTGAACTCCCTCTGTCCC
CAGGGCCTCCCTTCTGGGCCCCCTACAGCCCACCCTACCCCTCCTCCATGGGCCCTGCAGGAG
GGGAGACCCACCTTGAAGTGGGGGATCAGTAGAGGCTTGCACTGCCTTTGGGGCTGGAGGGA
GACGTGGGTCCACCAGGCTTCTGGAAAAGTCCTCAATGCAATAAAAACAATTTCTTTCTTGC

EMP

GGCCTCAACAAGGGAAACTTTATTGTTCCCGTGGGGCAGTCGAGGATGTCGGTGAATTACGCG
GCGGGGCTGTCGCCGTACGCGGACAAGGGCAAGTGCGGCCTCCCGGAGATCTTCGACCCCCC
GGAGGAGCTGGAGCGGAAGGTGTGGGAACTGGCGAGGCTGGTCTGGCAGTCTTCCAATGTGG
TGTTCCACACGGGTGCCGGCATCAGCACTGCCTCTGGCATCCCCGACTTCAGGGGTCCCCACG
GAGTCTGGACCATGGAGGAGCGAGGTCTGGCCCCCAAGTTCGACACCACCTTTGAGAGCGCG
CGGCCCACGCAGACCCACATGGCGCTGGTGCAGCTGGAGCGCGTGGGCCTCCTCCGCTTCCT
GGTCAGCCAGAACGTGGACGGGCTCCATGTGCGCTCAGGCTTCCCCAGGGACCAAACTGGCA
GAGCTCCACGGGAACATGTTTGTGGAAGAATGTGCCAAGTGTAAGACGCAGTACGTCCGAGA
CACAGTCGTGGGCACCATGGGCCTGAAGGCCACGGGCCGGCTCTGCACCGTGGCTAAGGCAA
GGGGGCTGCGAGCCTGCAGGGGAGAGCTGAGGGACACCATCCTAGACTGGGAGGACTCCCTG
CCCGACCGGGACCTGGCACTCGCCGATGAGGCCAGCAGGAACGCCGACCTGTCCATCACGCT
GGGTACATCGCTGCAGATCCGGCCCAGCGGGAACCTGCCGCTGGCTACCAAGCGCCGGGGAG
GCCGCCTGGTCATCGTCAACCTGCAGCCCACCAAGCACGACCGCCATGCTGACCTCCGCATCC
ATGGCTACGTTGACGAGGTCATGACCCGGCTCATGAAGCACCTGGGGCTGGAGATCCCCGCCT
GGGACGGCCCCCGTGTGCTGGAGAGGGCGCTGCCACCCCTGCCCCGCCCGCCCACCCCCAAG
CTGGAGCCCAAGGAGGAATCTCCCACCCGGATCAACGGCTCTATCCCCGCCGGCCCCAAGCA
GGAGCCCTGCGCCCAGCACAACGGCTCAGAGCCCGCCAGCCCCAAACGGGAGCGGCCCACC
AGCCCTGCCCCCCACAGACCCCCCAAAAGGGTGAAGGCCAAGGCGGTCCCCAGCTGACCAG
GGTGCTTGGGGAGGGTGGGGCTTTTTGTAGAAACTGTGGATTCTTTTTCTCTCGTGGTCTCACT
TTGTTACTTGTTTCTGTCCCCGGGAGCCTCAGGGCTCTGAGAGCTGTGCTCCAGGCCAGGGGT
TACACCTGCCCTCCGTGGTCCCTCCCTGGGCTCCAGGGGCCTCTGGTGCGGTTCCGGGAAGAA
GCCACACCCCAGAGGTGACAGCTGAGCCCCTGCCACACCCCAGCCTCTGACTTGCTGTGTTGT
CCAGAGGTGAGGCTGGGCCCTCCCTGGTCTCCAGCTTAAACAGGAGTGAACTCCCTCTGTCCC
CAGGGCCTCCCTTCTGGGCCCCCTACAGCCCACCCTACCCCTCCTCCATGGGCCCTGCAGGAG
GGGAGACCCACCTTGAAGTGGGGGATCAGTAGAGGCTTGCACTGCCTTTGGGGCTGGAGGGA
GACGTGGGTCCACCAGGCTTCTGGAAAAGTCCTCAATGCAATAAAAACAATTTCTTTCTTG

Supplementary Figure 2: RNA-seq of *Sirt6 mRNA* sequence of HUVEC or EMP. We found that the sequence of *Sirt6 mRNA* from HUVEC or EMP is uniform, therefore the *Sirt6 mRNAs* from HUVEC and EMP are mature and complete.
